# Congenital Goitre

**Published:** 1903-04-04

**Authors:** 


### CONGENITAL GOITRE.

An interesting point in connection with this rare affection is brought forward by Dr. Hewetson,[1] who remarks that in three of the reported cases it has been noted that the mother had been taking potassium chlorate during pregnancy. The first two of these cases were reported by Sir J. Y. Simpson in 1855, and Professor A. R. Simpson in 1886 respectively. The third occurred in the practice of Dr. G. A. Gibb, Birmingham, and is the one which Dr. Hewetson has been investigating. The maternal history is interesting, inasmuch as the mother had given birth to seven premature babies, none of which survived, during her six years of married life. No history of syphilis could be obtained, and nothing in the condition of the uterus was discoverable which might have accounted for the premature births. The infant affected with bronchocele was the seventh child. About the fourth week of pregnancy the mother commenced to take 12 grs. of potassium chlorate and 9 grs. of potassium iodide daily. This amount was slightly reduced when the child became viable, but was continued till the end of pregnancy. There was no history of goitre on either the father's or the mother's side. The tumour was about the size of a hen's egg, and consisted of an enlargement of the whole thyroid gland. On examination it showed an entire absence of cyst formation and colloid matter, together with marked vascularity of the tumour itself and increased size of the thyroid vessels. This, Dr. Hewetson says, is the commonest type of congenital bronchocele. The adenomatous and parenchymatous types of goitre may sometimes occur as congenital conditions. The three reported cases in which potassium chlorate had been given resembled each other in this, that all the mothers had been the subjects of numerous premature labours for which potassium chlorate had been given. Further experience and observation may prove whether or not the drug is a factor in the causation of congenital goitre.

[1] British Medical Journal, March 21.

